# Liver Transplant Patients with High Levels of Preoperative Serum Ammonia Are at Increased Risk for Postoperative Acute Kidney Injury: A Retrospective Study

**DOI:** 10.3390/jcm9061629

**Published:** 2020-05-28

**Authors:** Yoon Sook Lee, Yoon Ji Choi, Kyu Hee Park, Byeong Seon Park, Jung-Min Son, Ju Yeon Park, Hyun-Su Ri, Je Ho Ryu

**Affiliations:** 1 Department of Anaesthesiology and Pain Medicine, Ansan Hospital, Korea University, College of Medicine, Ansan 15355, Korea; yslee4719@gmail.com (Y.S.L.); pbskumc57@gmail.com (B.S.P.); 2 Department of Pediatrics, Korea University Hospital, Ansan 15355, Korea; czrabbit@korea.ac.kr; 3 Department of Biostatistics, Clinical Trial Center, Biomedical Research Institute, Pusan National University Hospital, Pusan 49241, Korea; statsjm@pnuh.co.kr; 4 Department of Anesthesiology and Pain Medicine, Daedong Hospital, Busan 47737, Korea; monojp@naver.com; 5 Department of Anaesthesia and Pain Medicine, Pusan National University Yangsan Hospital, Yangsan 50612, Korea; johnri@naver.com; 6 Division of Hepato-Biliary-Pancreatic Surgery and Transplantation, Department of Surgery, Pusan National University Yangsan Hospital, Pusan National University School of Medicine, Yangsan 50612, Korea; ryujhhim@hanmail.net

**Keywords:** acute kidney injury, ammonia, complication, liver transplantation

## Abstract

Acute kidney injury (AKI) is one of the most frequent postoperative complications after liver transplantation (LT). Increased serum ammonia levels due to the liver disease itself may affect postoperative renal function. This study aimed to compare the incidence of postoperative AKI according to preoperative serum ammonia levels in patients after LT. Medical records from 436 patients who underwent LT from January 2010 to February 2020 in a single university hospital were retrospectively reviewed. The patients were then categorized according to changes in plasma creatinine concentrations within 48 h of LT using the Acute Kidney Injury Network criteria. A preoperative serum ammonia level above 45 mg/dL was associated with postoperative AKI (*p* < 0.0001). Even in patients with a normal preoperative creatinine level, when the ammonia level was greater than 45 μg/dL, the incidence of postoperative AKI was significantly higher (*p* < 0.0001); the AKI stage was also higher in this group than in the group with preoperative ammonia levels less than or equal to 45 μg/dL (*p* < 0.0001). Based on the results of our research, an elevation in preoperative serum ammonia levels above 45 μg/dL is related to postoperative AKI after LT.

## 1. Introduction

The causes of postoperative acute kidney injury (AKI) after liver transplantation (LT) are multifactorial and include the status of the recipient and pre- and post-operative risk factors [1]. Despite advances in organ preservation and surgery techniques and improvements in immunosuppression protocols, there are studies that still report high incidence rates of AKI, even up to 50%, after LT [2,3]. The development of postoperative AKI in the first year after LT is a major factor influencing long-term survival [4,5]. In addition, postoperative AKI can potentially progress to a requirement for post-transplant kidney replacement therapy [6], to chronic kidney disease, and/or progress to end-stage renal disease [4,7]. In reality, 18.1% of patients showed progression to chronic kidney disease after LT, and 4.8% of patients showed progression to end-stage renal disease within 5 years [8,9].

Pre-operative renal dysfunction often affects the degree of liver dysfunction and significantly affects the development of postoperative AKI [10,11]. Failure to manage adequate intravascular volume during surgery, surgery-related events, increased use of high-risk or marginal grafts, hemodynamic instability, severity of post-reperfusion syndrome including hepatic ischemia-reperfusion injury, and use of nephrotoxic medications can further increase the risk of AKI incidence [1,12]. In particular, liver disease often affects postoperative renal function due to the nature of the disease, which can include the following: a history of ascites, encephalopathy, hepato-renal syndrome, spontaneous bacterial peritonitis, and hypoalbuminemia [13,14]. Elevation of serum ammonia levels, which occurs during liver disease, may also affect kidney function.

In healthy individuals, ammonia is converted to glutamine and urea by the liver using circulating urea and citric acid, preventing it from entering the systemic circulation. Urea is transported through the bloodstream and excreted by the kidneys into the urine [5]. However, as liver disease progresses, blood ammonia levels increase as a result of liver dysfunction, and blood is shunted around the liver. In addition, since muscle is an important site for removing extrahepatic ammonia, muscle wasting also contributes to increased ammonia levels in advanced liver disease [15]. This increase in serum ammonia levels due to problems with the liver itself, and not due to reduced excretion owing to kidney damage, is likely to affect postoperative renal function. The increased systemic ammonia concentration can directly influences glomerular cells, contributing to glomerular damage and tubular fibrosis [16,17,18].

Therefore, in patients undergoing liver transplantation, there is a need to be concerned about the adverse role of high levels of serum ammonia on renal function after surgery. The purpose of our study was to investigate whether preoperative serum ammonia levels could predict the incidence of postoperative AKI after LT.

## 2. Experimental Section

### 2.1. Patient Population

**Figure 1:**
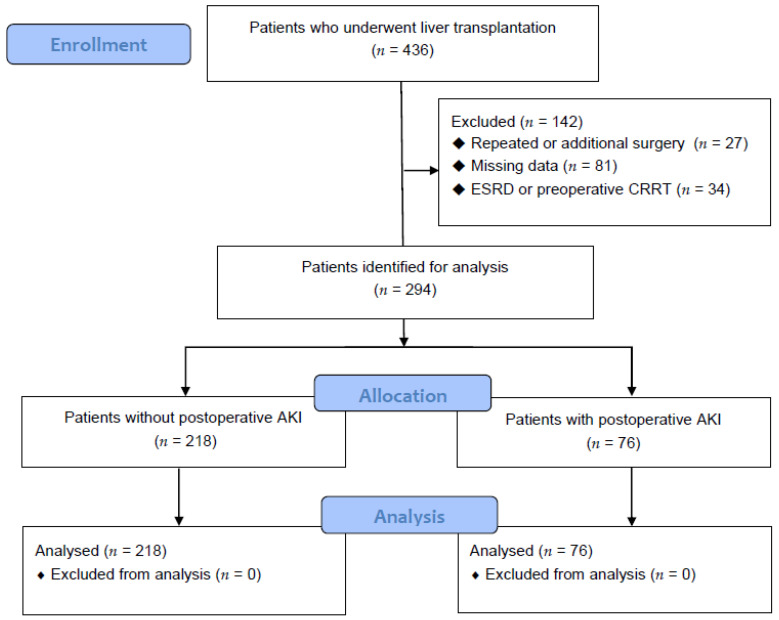
Flow diagram analyzing the effect of ammonia on postoperative acute kidney injury in liver transplant patients.

This study was approved by the hospital clinical research committee (IRB No. 05-2019-032) and the requirement for written informed consent was waived by the Institutional Review Board. The computerized medical record data of 436 patients who underwent LT from January 2010 to February 2020 were collected (Figure 1). This study excluded 142 patients who had undergone re-transplantation or an additional surgery, had end-stage renal disease or were receiving continuous renal replacement therapy preoperatively, or had missing data.

### 2.2. Data Collection

In this study, patients were divided into 2 groups: those without postoperative AKI (Group C, *n* = 218) and those with postoperative AKI (Group AKI, *n* = 76). Data on the patients’ basic demographic characteristics, laboratory test results, and treatments were collected before, during, and after the operation. Patient records, preoperative evaluation records, anesthesia records, intensive care unit records, progress notes, hospital nursing records, cooperative records, and discharge records were reviewed retrospectively. The perioperative use of anesthesia was also assessed. A preoperative laboratory examination was defined as a test performed within 24 h prior to surgery. A postoperative laboratory test was defined as a test performed within 24 h of the operation.

### 2.3. Intraoperative Protocol

LT and anesthesia were performed using conventional methods. The recipient hepatectomy was performed after a cholecystectomy and total hepatectomy. A donor liver was inserted, and portal vein anastomosis was performed. After blood was re-perfused through the portal vein, hepatic artery anastomosis was performed and re-perfused. Biliary reconstruction and bleeding control were then performed. Finally, the abdomen was closed. None of the final enrolled patients underwent a piggyback technique or a venovenous bypass.

All patients underwent standard monitoring with electrocardiography; the parameters of end-tidal carbon dioxide concentration, bispectral index, peripheral oxygen saturation, and cerebral blood oxygenation were monitored. Invasive arterial monitoring using the radial and femoral arteries was also applied. Central venous oxygen saturation monitoring (PreSep, Edwards Lifesciences, Irvine, CA, USA) was performed using an EV1000 monitoring platform (Edwards Lifesciences) to monitor stroke volume, stroke volume index, cardiac output, cardiac index, central venous oxygen saturation, and systemic vascular resistance.

General anesthesia was induced using 1–2 mg/kg of propofol and muscle relaxants such as 0.6–1 mg/kg of rocuronium or 0.1–0.2 mg/kg of cisatracurium. Inhaled anesthetic gases of sevoflurane or desflurane were treated with an oxygen/air mixture of 40–50% fraction of inspired oxygen. Remifentanil (range, 0.5–10 mcg/kg/h) and muscle relaxants (rocuronium: range, 0.3–0.6 mg/kg/h; or cisatracurium: range, 0.1–0.2 mg/kg/h) were infused during the operation.

The transfusion protocol was performed according to the patient’s condition such that the hematocrit level could be maintained at 25–30%. Norepinephrine (0.01–0.4 mcg/kg/min) was administered to maintain systolic blood pressure and mean arterial pressure above 90 and 60 mmHg, respectively. If norepinephrine was not effective, dobutamine, vasopressin, or epinephrine were considered.

After the completion of surgery, the patient was moved to the intensive care unit (ICU). When the patient’s vital signs were stabilized with no bleeding and if the patient could recover consciousness and spontaneous breathing, extubation was performed.

### 2.4. Definition of AKI

AKI in the postoperative period was diagnosed according to the Acute Kidney Injury Network criteria and was based on changes in serum creatinine (Cr) concentrations within 48 h of surgery [7]. Stage I was defined as an increase of ≥0.3 mg/dL in serum Cr levels from baseline (normal reference range, 0.7–1.4 g/dL at our institution) or a 150–200% increase from baseline, stage II was defined as a 200–300% increase from baseline, and stage III was defined as an increase of >300% from baseline, a serum Cr level ≥4.0 mg/dL accompanied by an acute increase of ≥0.5 mg/dL from baseline, or a need for renal replacement therapy irrespective of other criteria.

### 2.5. Statistical Analysis

Data were analyzed using Statistical Analysis System version 9.3 (SAS Institute, Cary, NC, USA) and R software version 3.3.2 (R Project for Statistical Computing, Vienna, Austria) [19].

Data were expressed as the mean ± standard deviation, median (25–75th percentile), or number of patients (%). Normality testing was performed using the Shapiro–Wilk W or Kolmogorov–Smirnov test. The independent t-test or Wilcoxon rank-sum test was used for comparing continuous variables between the two groups. The χ^2^ test or Fisher’s exact test was used for comparing categorical variables. A *p*-value < 0.05 was considered statistically significant.

A receiver operating characteristic curve analysis was performed to assess the significance of the relationship between preoperative ammonia levels and postoperative AKI after LT. Sensitivity and specificity were established to use the potential cut-off values to distinguish the association between postoperative AKI and serum ammonia levels.

The effect of preoperative ammonia levels on the incidence of postoperative AKI was assessed using univariate and multivariable analyses. The typical factors associated with postoperative AKI after LT were analyzed using univariate analysis, and multivariable analysis was performed using 6 factors with a *p*-value < 0.05.

Additionally, the effects of preoperative ammonia levels on postoperative AKI after LT in patients with normal preoperative serum Cr levels and elevated preoperative serum Cr levels were analyzed using the χ^2^ test or Fisher’s exact test. The normal cutoff value for serum Cr in our hospital is 1.2 mg/dL.

## 3. Results

A total of 436 patients were analyzed (Figure 1), and 218 and 76 patients were categorized into Group C and Group AKI, respectively. In this study, 76/294 (25.85%) patients had postoperative AKI after LT.

The characteristics of the patients who underwent LT are shown in Table 1. Patients in Group AKI were older (*p* = 0.032), but there was no difference in sex or body mass index. AKI was more common in those receiving transplants from cadaver donors than in those receiving transplants from living donors (*p* < 0.001). The model for the end-stage liver disease scores was higher in patients in Group AKI than those in Group C (*p* < 0.001).

The preoperative laboratory data of the patients who underwent LT are shown in Table 2. Lower preoperative hemoglobin levels (*p* = 0.004), higher preoperative ammonia levels (*p* < 0.001), higher preoperative bilirubin levels (*p* = 0.001), and higher prothrombin time (international normalized ratio) were seen in Group AKI than in Group C (*p* < 0.0001).

The perioperative factors of the patients who underwent LT are shown in Table 3. The AKI group had shorter anesthesia time (*p* = 0.013) but had higher estimated blood loss (*p* < 0.001), total fluid intake (*p* = 0.006), and number of transfusions (*p* = 0.001) than the C group.

Postoperative data of the patients who underwent LT are shown in Table 4. There was no significant difference in postoperative hemoglobin levels between the groups (*p* = 0.633), but postoperative bilirubin levels were higher in Group AKI (*p* = 0.007). Additionally, protein levels (*p* = 0.018) were lower and Cr levels were higher (*p* < 0.001) in Group AKI than in Group C. The duration of stay in the ICU (*p* < 0.0001) and the time between surgery and discharge (*p* = 0.006) were longer in Group AKI than in Group C.

**Figure 2:**
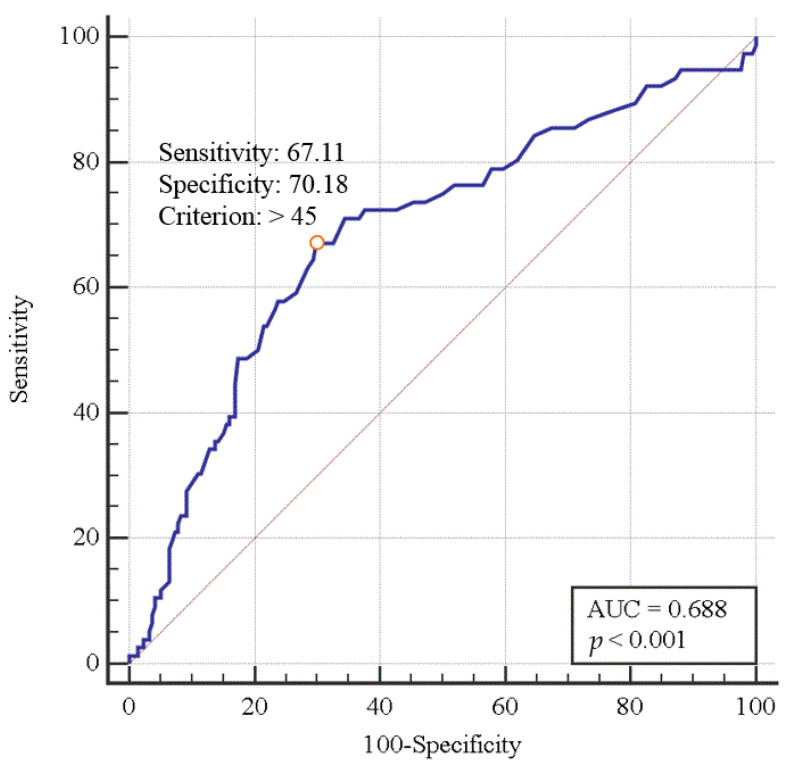
Diagnostic performance (receiver operating characteristic curve) of serum ammonia level for predicting the incidence of postoperative acute kidney injury in liver transplant patients.

The cutoff value of the preoperative serum ammonia level was shown to be 45 mg/dL, with a sensitivity of 67.11% and a specificity of 70.18% (area under the curve, 0.688; 95% confidence interval, 0.632–0.741; *p* < 0.0001; Figure 2) for predicting postoperative AKI.

The univariate analysis showed that ammonia levels (>45 μg/dL), age, model for end-stage liver disease score, preoperative serum hemoglobin level, total fluid intake, and transfused packed red blood cells were related to postoperative AKI after LT, as shown in Table 5. The multivariable analysis revealed that preoperative ammonia levels (>45 μg/dL; *p* < 0.0001) and age (*p* = 0.034) were independent risk factors for postoperative AKI after LT.

Additionally, the effect of preoperative ammonia levels on postoperative AKI after LT in patients with normal preoperative serum Cr levels and elevated preoperative serum Cr levels are shown in Table 6. For patients after LT, when ammonia levels were greater than 45 mg/dL, postoperative AKI occurred more frequently (*p* < 0.0001), and the AKI stage was higher than that in the group with serum ammonia levels less than or equal to 45 mg/dL (*p* < 0.0001). Furthermore, for patients with a normal preoperative Cr level, when serum ammonia levels were greater than 45 mg/dL, the incidence and stage of postoperative AKI were significantly higher (*p* < 0.001) than those in the group with serum ammonia levels less than or equal to 45 mg/dL (*p* < 0.0001). For patients with elevated preoperative serum Cr levels, there was no significant difference in the incidence of postoperative AKI after LT between the two groups (*p* = 0.321), but the stage of postoperative AKI was significantly higher (*p* < 0.0001) in patients with ammonia levels greater than 45 mg/dL.

## 4. Discussion

A preoperative serum ammonia level above 45 μg/dL was correlated with postoperative AKI after LT in this retrospective analysis of LT recipients. When ammonia levels were greater than 45 μg/dL, postoperative AKI occurred more frequently, and the AKI stage was higher than that in the group with serum ammonia levels less than or equal to 45 μg/dL. Additionally, even in patients with normal preoperative Cr levels, the incidence of postoperative AKI and the AKI stage were significantly higher in the group with preoperative ammonia levels greater than 45 μg/dL than in the group with ammonia levels of 45 μg/dL or less.

Postoperative AKI is one of the most frequent complications after LT, and it is associated with poor results of transplantation and worse outcomes, including increased mortality and morbidity, longer ICU and hospital stays, lower graft survival, increased postoperative infection rates, and a higher incidence of chronic renal disease [20,21,22,23]. The incidence of AKI varies from 17.0% to 95.0% in patients undergoing orthotopic LT [20], and from 29.0% to 63.1% in patients undergoing living donor LT [21,22,23], depending on the definition adopted. In 38 cohort studies of 13,422 LT patients, the estimated incidence rates of severe AKI requiring the estimated incidence of AKI and renal replacement therapy after LT were 40.7% and 7.7%, respectively [24]. The incidence of AKI is high after LT because many factors associated with the nature of LT can affect renal function. The development of postoperative AKI after LT appears to be multifactorial, with the involvement of many preoperative, perioperative, and postoperative factors. End-stage liver disease itself can be accompanied by renal dysfunction [24,25]. Physiological changes characterized by end-stage hepatic failure are primarily associated with an underlying disease or are secondary to the development of hepatorenal syndrome [25]. In addition, LT recipients face a number of risk factors for kidney damage during the perioperative period, including intraoperative bleeding, graft dysfunction, postoperative sepsis, and nephrotoxicity of calcineurin inhibitors, all of which may contribute to AKI [24,26]. While maintaining adequate blood pressure values and renal perfusion has a potentially favorable impact on the prevention of AKI, in liver transplant surgery, strategies are implemented to adopt fluid restriction and maintain low central venous pressure in order to reduce intraoperative blood loss and to achieve an optimal surgical field. This approach may thus carry an intrinsically high risk of decreasing renal perfusion and increasing the risk of AKI [27]. In addition, the massive blood loss during surgery with or without an increased renal susceptibility to ischemia and intraoperative red blood cell transfusion are risk factors for postoperative AKI [27,28,29] and require rigorous perioperative control of the patient hemodynamic status.

An increase in serum ammonia levels is one characteristic of end-stage liver disease. Generally, ammonia is one of the main products of nitrogen metabolism and is usually transported from the muscles and other peripheral tissues to the liver, converted into urea by the urea cycle, and excreted by the kidneys via urine [30]. When acute or chronic liver disease develops, the liver’s ability to synthesize urea is impaired due to a disorder in the main pathway of ammonia detoxification [15,31]. Intrahepatic and extrahepatic portosystemic shunting, which is caused by an exacerbation of liver disease, causes hyperammonemia [15]. In addition, in chronic liver disease, kidney dysfunction often appears in the form of hepatorenal syndrome. This is a situation where an increase in cardiac output and splanchnic vasodilatation leads to a decrease in renal perfusion and the glomerular filtration rate. As a result, the levels of angiotensin II and antidiuretic hormone increase, which increases renal vasoconstriction, worsening renal perfusion and kidney function [27,32,33]. This exacerbates the increase in serum ammonia levels.

In healthy people, when hyperammonemia occurs, the net ammonia in the systemic circulation is collected by the kidneys [34]. However, in patients with cirrhosis, the kidneys are the main source of ammonia. The increased systemic ammonia concentration directly influences glomerular cells, contributing to glomerular damage and tubular fibrosis [16,17,18]. Hyperammonemia leads to the progression of kidney injury through the stimulating effect of ammonia on renal growth as well as the complement cascade. Additionally, oxidative stress can cause renal ammonia production, contributing to the progression of kidney damage [35]. Moreover, in acute hepatic failure, the N-methyl D aspartate receptor is activated, which contributes to kidney damage caused by hyperammonemia [36]. Ammonium salts in the kidneys causes cortical and coronary necrosis, and necrotic kidneys show a decrease in Cr clearance [37,38]. Overall, these findings suggest that hyperammonemia leads to kidney dysfunction and continued kidney damage.

The main limitation of this study resides in its retrospective design. As the patients who developed AKI in the postoperative period had more severe liver disease at baseline, and more perioperative complications, we performed multivariable analyses to adjust for many factors potentially associated with the risk of developing AKI after LT. However, the presence of further unmeasured confounders is likely, and thus, the results of our study should be regarded as hypothesis-generating.

## 5. Conclusions

In conclusion, when preoperative serum ammonia levels are greater than 45 mg/dL in patients undergoing LT, the development of postoperative AKI should be carefully considered, and managements should be taken for its prevention. In addition, even when the preoperative renal function was normal, postoperative AKI after LT was more common when serum ammonia levels were above 45 mg/dL; the stage of AKI was also higher.

## Figures and Tables

**Table 1:** Characteristics of patients undergoing liver transplantation.

	C Group (*n* = 218)	AKI Group (*n* = 76)	*p*-Value
Age (yr)	52.72 ± 8.49	55.09 ± 7.50	0.032 *
Sex (F/M)	156 (71.56)/62 (28.44)	55 (72.37)/21 (27.63)	0.893
Body mass index (kg/m^2^)	22.05 ± 3.19	22.76 ± 3.66	0.108
CDLT/LDLT	62 (28.44)/156 (71.56)	40 (52.63)/36 (47.37)	<0.001 *
Cause of LT			0.007 *
Alcoholic cirrhosis	46 (21.1)	22 (28.95)	
HBV/HCV infection	149 (68.35)	37 (48.68)	
NBNC LC	11 (5.05)	5 (6.58)	
Toxic hepatitis	8 (3.67)	10 (13.16)	
Primary biliary cirrhosis	4 (1.83)	2 (2.63)	
MELD score	17.52 ± 11.06	22.20 ± 10.48	0.001 *
Hypertension	43 (19.72)	15 (19.74)	0.998
Diabetes mellitus	53 (24.31)	22 (28.95)	0.425
Ischemic heart disease	1 (0.46)	1 (1.32)	0.451
Congestive heart failure	1 (0.46)	2 (2.63)	0.165
Cerebrovascular accidents	2 (0.92)	0 (0.00)	1.000
Cardiac arrhythmia	5 (2.29)	3 (3.95)	0.430
With diuretics	46 (21.1)	14 (18.42)	0.618
With insulin	8 (3.67)	4 (5.26)	0.515
With ß-blocker	8 (3.67)	2 (2.63)	1.000
With calcium channel blocker	14 (6.42)	2 (2.63)	0.256

Values are presented as mean ± standard deviation or number (%). C group: patients without postoperative AKI after LT, AKI group: patients with postoperative AKI after LT. AKI: acute kidney injury, CDLT: cadaveric donor liver transplantation, LDLT: living donor liver transplant, LT: liver transplantation, HBV: hepatitis B virus, HCV: hepatitis C virus, NBNC LC: non-HBV non-HCV liver cirrhosis, and MELD: Model for End-Stage Liver Disease. * *p* < 0.05 compared between groups.

**Table 2:** Preoperative laboratory data of patients undergoing liver transplantation.

	C Group (*n* = 218)	AKI Group (*n* = 76)	*p*-Value
Hemoglobin (g/dL)	11.04 ± 2.34	10.17 ± 2.07	0.004 *
Platelets (×10^3^/mL)	66.50 (46.00, 106.00)	57.00 (43.67, 81.25)	0.067
Creatinine (mg/dL)	0.77 (0.63, 0.98)	0.84 (0.63, 1.00)	0.492
AST (unit/L)	50.50 (34.00, 76.00)	45.50 (34.00, 80.75)	0.474
ALT (unit/L)	31.00 (21.25, 50.00)	27.00 (17.00, 43.75)	0.113
Protein (g/dL)	6.01 ± 0.87	5.96 ± 0.90	0.652
Albumin (g/dL)	3.19 ± 0.58	3.06 ± 0.53	0.085
Ammonia (μg/dL)	35.50 (24.00, 49.00)	55.50 (35.75, 76.25)	<0.001 *
≤45 μg/dL	153 (70.2)	25 (32.9)	<0.001 *
>45 μg/dL	65 (29.8)	51 (67.1)	
Bilirubin (mg/dL)	2.40 (1.22, 12.02)	4.40 (2.18, 15.57)	0.001 *
PT (INR)	1.47 (1.21, 2.10)	1.91 (1.54, 2.43)	<0.001 *
Sodium (mEq/L)	136.49 ± 5.63	136.75 ± 5.38	0.722
Potassium (mEq/L)	3.93 ± 0.49	4.04 ± 0.52	0.086

Values are expressed as mean ± standard deviation or median (interquartile range). C group: patients without postoperative AKI after LT, AKI group: patients with postoperative AKI after LT, AKI: acute kidney injury, AST: aspartate aminotransferase, ALT: alanine aminotransferase, and PT (INR): prothrombin time (international normalized ratio). * *p* < 0.05 compared between groups.

**Table 3:** Perioperative factors of patients undergoing liver transplantation.

	C Group (*n* = 218)	AKI Group (*n* = 76)	*p*-Value
Anesthesia duration (h)	11.00 (9.50, 12.50)	10.00 (8.50, 12.00)	0.013 *
Estimated blood loss (L)	2.50 (1.50, 4.50)	3.35 (2.50, 6.00)	<0.001 *
Total fluid intake (L)	6.90 (5.18, 9.67)	8.07 (6.08, 12.23)	0.006 *
pRBC (unit)	4.00 (0.00, 8.00)	7.00 (2.00, 12.00)	0.001 *
Fresh frozen plasma (unit)	4.00 (0.00, 9.00)	7.50 (2.00, 12.00)	0.001 *
Cryoprecipitate (unit)	0.00 (0.00, 0.00)	0.00 (0.00, 0.00)	0.428
Platelets (unit)	0.00 (0.00, 0.00)	0.00 (0.00, 8.00)	0.001 *

Values are expressed as median (interquartile range). C group: patients without postoperative AKI after LT, AKI group: patients with postoperative AKI after LT, AKI: acute kidney injury, and pRBC: packed red blood cells. * *p* < 0.05 compared between groups.

**Table 4:** Postoperative data of patients who underwent liver transplantation.

	C Group (*n* = 218)	AKI Group (*n* = 76)	*p*-Value
Hemoglobin (g/dL)	9.04 ± 1.45	8.94 ± 1.58	0.633
Platelets (×10^3^/mL)	53.50 (39.25, 77.00)	46.50 (36.00, 61.50)	0.053
PT (INR)	1.76 (1.54, 1.95)	1.80 (1.42, 2.04)	0.919
Bilirubin (mg/dL)	4.10 (2.10, 6.68)	5.75 (2.98, 8.65)	0.007 *
Protein (g/dL)	5.30 ± 0.65	5.09 ± 0.74	0.018 *
Albumin (g/dL)	3.66 ± 0.45	3.56 ± 0.56	0.160
Creatinine (mg/dL)	0.90 ± 0.33	1.39 ± 0.48	<0.001 *
Sodium (mEq/L)	139.30 ± 3.71	140.12 ± 4.23	0.111
Potassium (mEq/L)	3.96 ± 0.38	4.12 ± 0.47	0.007 *
Stay in the intensive care unit (days)	7.00 (5.00, 11.00)	11.00 (6.00, 17.50)	<0.001 *
Time from surgery to discharge (days)	27.00 (22.00, 38.00)	32.50 (25.00, 52.50)	0.006 *

Values are expressed as the mean ± standard deviation, number (%), or median (range). C group: patients without postoperative AKI after LT, AKI group: patients with postoperative AKI after LT, AKI: acute kidney injury, and PT (INR): prothrombin time (international normalized ratio). * *p* < 0.05 compared between groups.

**Table 5:** Logistic regression analysis in patients undergoing liver transplantation.

Predictors	OR	95% CI		*p*-Value *	OR	95% CI		*p*-Value †
		Lower	Upper			Lower	Upper	
Ammonia (>45)	4.802	2.744	8.403	<0.0001 *	4.081	2.242	7.427	<0.0001 †
Preoperative patient characteristics								
Age	1.038	1.003	1.075	0.033 *	1.040	1.003	1.079	0.034 †
Sex	1.041	0.581	1.863	0.893				
Body mass index	1.066	0.986	1.153	0.109				
MELD score	1.037	1.014	1.062	0.002 *	1.010	0.980	1.041	0.531
Hypertension	1.001	0.519	1.929	0.998				
Diabetes mellitus	1.268	0.707	2.275	0.425				
IHD	2.889	0.178	46.766	0.455				
CHF	5.864	0.524	65.604	0.151				
With ß-blocker	0.394	0.087	1.774	0.225				
With CCB	0.710	0.147	3.417	0.669				
With diuretics	0.844	0.434	1.642	0.618				
With insulin	1.459	0.426	4.988	0.547				
Preoperative laboratory data								
Hemoglobin	0.736	0.639	0.849	<0.0001 *	0.921	0.789	1.074	0.295
Platelets	0.994	0.989	1.000	0.069				
Bilirubin	1.016	0.996	1.037	0.124				
Albumin	0.660	0.410	1.061	0.086				
Na	1.009	0.962	1.057	0.721				
K	1.590	0.934	2.707	0.087				
Cr	1.100	0.649	1.866	0.723				
Intraoperative data								
Anesthesia duration	0.899	0.805	1.003	0.058				
EBL	1.070	0.993	1.152	0.075				
Total fluid	1.046	1.003	1.091	0.038 *	0.992	0.932	1.055	0.798
pRBC	1.051	1.014	1.090	0.007 *	1.020	0.974	1.068	0.394

OR: odds ratios, CI: confidence interval, MELD: Model for End-Stage Liver Disease, IHD: Ischemic heart disease, CHF: Congestive heart failure, CCB: calcium channel blocker, Na: Sodium, K: potassium, Cr: creatinine, EBL: estimated blood loss, and pRBC: packed red blood cells. * *p*-value of univariate analysis <0.05. † *p*-value of multivariate analysis <0.05.

**Table 6:** Incidence of AKI according to ammonia levels in patients undergoing liver transplantation.

	Overall (*n* = 294)	≤45 μg/dL (*n* = 178)	>45 μg/dL (*n* = 116)	*p*-Value
Total				<0.0001 *
Normal	218 (74.15)	153 (85.96)	65 (56.03)	
AKI	76 (25.85)	25 (14.04)	51 (43.97)	
AKI stage				<0.0001 *
0	218 (74.15)	153 (85.96)	65 (56.03)	
1	62 (21.09)	21 (11.80)	41 (35.34)	
2	11 (3.74)	3 (1.69)	8 (6.90)	
3	3 (1.02)	1 (0.56)	2 (1.72)	
Creatinine ≤ 1.2 mg/dL				
Normal	185 (74.9)	140 (86.4)	45 (52.9)	<0.001 *
AKI	62 (25.1)	22 (13.6)	40 (47.1)	
AKI stage				
0	185 (74.9)	140 (86.4)	45 (52.9)	<0.001 *
1	48 (19.4)	18 (11.1)	30 (35.3)	
2	11 (4.5)	3 (1.9)	8 (9.4)	
3	3 (1.2)	1 (0.6)	2 (2.4)	
Creatinine > 1.2 mg/dL				
Normal	33 (70.2)	13 (81.2)	20 (64.5)	0.321
AKI	14 (29.8)	3 (18.8)	11 (35.5)	
AKI stage				<0.0001 *
0	218 (74.15)	153 (85.96)	65 (56.03)	
1	62 (21.09)	21 (11.80)	41 (35.34)	
2	11 (3.74)	3 (1.69)	8 (6.90)	
3	3 (1.02)	1 (0.56)	2 (1.72)	

AKI: Acute kidney injury. * *p* < 0.05 compared between groups.
